# Internet Addiction among Secondary School Adolescents: A Mixed Methods Study

**DOI:** 10.31729/jnma.4292

**Published:** 2019-10-31

**Authors:** Karma Tenzin, Thinley Dorji, Tshering Choeda, Phuntsho Wangdi, Myo Minn Oo, Jaya Prasad Tripathy, Tashi Tenzin, Tashi Tobgay

**Affiliations:** 1 Faculty of Postgraduate Medicine, Khesar Gyalpo University of Medical Sciences of Bhutan, Thimpu, Bhutan; 2 Armed Force Medical College, Pune, India; 3 Ministry of Education, Thimphu, Bhutan; 4 International Union against Tuberculosis and Lung Disease, Myanmar; 5 Department of Community Medicine, All India Institute of Medical Sciences, Nagpur, India; 6 World Health Organisation, Thimphu, Bhutan

**Keywords:** *adolescent*, *anxiety*, *depression*, *internet addiction*, *stress*

## Abstract

**Introduction:**

Excessive use of internet can lead to “Internet Addiction”. A sharp rise in the number of internet users among adolescents in Bhutan have been observed lately which could have potential side-effects on their health. The aim of this study was to find the prevalence of internet addiction and psychological co-morbidities among the secondary school-going adolescents.

**Methods:**

This is a descriptive cross-sectional quan-qual sequential mixed method design with a quantitative component and qualitative component. The study was conducted between 1^st^ May and 30^st^ November 2017 conducted in twelve schools across Bhutan after taking ethical clearance was from Research Ethics Board of Health of Bhutan. Data were double-entered in EpiData Entry, validated and imported into STATA version 12.0 for analysis. Point estimate at 95% CI was calculated along with frequency and proportion for binary data.

**Results:**

Among 721 adolescents from twelve selected schools, prevalence of internet addiction was 248 (34.440%) at, 95% Confidence Interval (31-38%). Out of A total of 586 (81%) preferred smartphone for internet use with 388 (54%) actually using it. Depression and anxietywere the associated psychological co-morbidities seen along with internet addiction. Internet addiction was high among those using internet at home, using smartphone for internet access, social networking and night time internet use were associated with internet addiction. Boredom, stress/anxiety and peer pressure were triggers of internet use. Internet addiction also affected academic performance, social interactions and sleep.

**Conclusions:**

Prevalence of internet addiction among adolescents in Bhutan is high comparing to previous standard data which requires immediate attention.

## INTRODUCTION

Excessive use of internet can lead to “Internet Addiction” (IA) which has been defined as internet use that interferes with daily life.^1^ IA also known as pathological or addictive ilnternet use was first recognized as a health issue in the United States in the mid-1990s.^2^

The majority of the internet users are found to be adolescents and young adults.^3,4^ A review of literature shows that compulsive and heavy ilnternet use has been associated with potential detrimental side-effects, such as depression and suicidal ideation, loneliness, interpersonal problems, poor school performance and personal relationships, time management problems, sleeplessness, unhealthy lifestyles and poor dietary behaviours.^5,6^

Recent unpublished report on internet addiction among the college students in Bhutan found high prevalence of internet addiction at 36%. However, there are no estimates of internet addiction and its associated factors among the school-going adolescents in Bhutan.

The aim of this study was to find the prevalence of internet addiction and psychological co-morbidities among the secondary school-going adolescents.

## METHODS

This is a descriptive cross-sectional quan-qual sequential mixed method design with a quantitative component and a descriptive qualitative component. The study was conducted between 1^st^ May and 30^st^ November 2017 in twelve selected higher secondary schools across Bhutan.

For the quantitative component, the study population included a sample of secondary school-going of adolescents of age group 11-19 years of class IX-XII in twelve selected schools of Bhutan. For the qualitative component, the study population included secondary school-going adolescents those who were found to have internet addiction test score more than 49.

Ethical approval was taken from the Union Ethics Advisory Group, Paris, France (Approval no. EAG no. 34/17) and Research Ethics Board of Health in Bhutan (Approval no. REBH/Approval/2017/029).

Quantitative component

A stratified multi-stage sampling was adopted to achieve the required sample size. The first stage sampling unit included schools while the second stage sampling unit included students selected from each school. A list of all eligible higher secondary schools was prepared and stratified by rural-urban location. A total of 6 urban (secondary) and 6 rural (central) schools were randomly selected from a list of these schools. From each school, a list of students studying in class IX-XII was obtained and a total of around 55 students were selected by systematic random sampling after calculating the sampling interval (total number of students in class IX-XII/55).

Assuming the prevalence of internet addiction among adolescents to be 30%,^7^ absolute precision of 3.5%, and a sample size of 659 was estimated. Sample size was calculated by the following formula,

n = Z^2^ × p × q/e^2^

   = 3.84 × (0.3 × 0.7)/0.035^2^

   = 659

where,

n = minimum sample size
Z = 1.96 for 95% confidence intervals
p = prevalence from previous study (30%)
q = 1-p
e = Margin of error (for example, 0.035 for 3.5%)

However, to account for loss due to non-response of 9%, it was decided to recruit a total of at least 721 students in the study.

The selected students were interviewed using a self-administered questionnaire. The questionnaire had 3 parts, Part I: socio-demographic characteristics and pattern of internet use; Part II: Validated Internet Addiction Test (IAT) questionnaire,^8^ and Partlll: Validated Depression, Anxiety and Stress Scales (DASS 21).^8,9^

Data were collected by a self-administered questionnaire in every school. In every school, a health teacher or counselor was trained regarding the process of data collection and administration of the questionnaire. The list of systematically sampled students studying in Class IX-XII was shared with the school teacher/counselor. Data collection was conducted in classroom settings.

Selection bias, information bias and self-reporting bias has been minimized. Data were double-entered in EpiData Entry, validated and imported into STATA version 12.0 for analysis. Point estimate at 95% CI was calculated along with frequency and proportion for binary data.

Qualitative component

Internet addiction was operationally defined in this study as a person whose IAT score>49 points (i.e. a combination of moderately and severely addicted.^8,9^

As part of qualitative component, total of 13 students were conveniently selected among those who were found to be addicted to internet (i.e. score of>49 on IAT) and those who consented for the interview. Saturation of findings guided the size of the sample.

Those students who scored>49 on the IAT and willing to participate were recruited for in-depth interviews (IDIs). The interviews were conducted by the Dr. Karma Tenzin, trained in qualitative research with more than 5 years of research experience (MBBS, MD). The investigator was a well versed in local Bhutanese language. The IDIs were conducted at a time and place convenient to the participants using a topic guide preferably in a room with adequate privacy. Each interview roughly lasted around 15-20 minutes. Only the researcher and the participant were present during the interview, which was done to avoid biased responses in the presence of the school teacher.

The primary investigator noted the proceedings of the interviews and transcribed on the day of the interview in English. The interviews were audio recorded (n=6) wherever consent was provided. A manual content analysis of the transcripts was done by the study investigators (Jaya Prasad Tripathy and Myo Minn Oo). The transcripts were then reviewed by another investigator (Karma Tenzin) to reduce subjectivity in interpretation. Any difference between the investigators was resolved by discussion and referral back to the original transcripts/audio files where necessary. Codes were generated and combined into themes using standard procedures and in consensus.^10^ The themes have been described along with verbatim quotes (written in italics where square brackets denote author’s statements). The guidelines under “Consolidated Criteria for Reporting Qualitative Research” was used to report the study findings.^11^

Data were double-entered in EpiData Entry (version 3.1), EpiData Association, Odense, Denmark), validated and imported into STATA version 12.0 for analysis.

## RESULTS

A. Quantitative

Out of a total of 721 school going adolescents, prevalence of moderate and severe internet addiction was seen among 237 (32.9%) at 95% of CI (29.5-36.4) and 11 (1.5%) at 95% of CI: (0.9-2.7) adolescents respectively. Severe to extremely severe depression, anxiety and stress was found in 82 (11.4%), 205 (28.4%) and 42 (5.9%) of them respondents respectively (Table 1).

**Table 1:** Internet addiction, depression, anxiety and stress among secondary school-going adolescents (n=721).

Characteristics	n (%)	95% Cl
Internet Addiction		
Normal/Mild	371 (51.5)	(47.8-55.1)
Moderate	237 (32.9)	(29.5-36.4)
Severe	11 (1.58)	(0.9-2.7)
Missing	102(14.1)	(11.8-16.9)
Mental Wellbeing		
Depression		
Normal	306 (42.4)	(38.9-46.1)
Mild	128(17.8)	(15.1-20.7)
Moderate	182 (25.2)	(22.2-28.5)
Severe	51 (7.1)	(5.4-9.2)
Extremely severe	31 (4.3)	(3.0-6.0)
Missing	23 (3.2)	(2.1-4.7)
Anxiety		
Normal	231 (32.0)	(28.7-35.5)
Mild	67 (9.37)	(7.4-11.6)
Moderate	186(25.8)	(22.7-29.1)
Severe	85(11.8)	(9.6-14.4)
Extremely severe	120(16.6)	(14.1-19.5)
Missing	32 (4.4)	(3.2-6.2)
Stress		
Normal	455(63.1)	(59.5-66.6)
Mild	84(11.6)	(9.5-14.2)
Moderate	93(12.9)	(10.6-15.5)
Severe	33 (4.6)	(33-6.4)
Extremely severe	9(1.3)	(0.7-2.4)
Missing	47 (6.5)	(4.9-8.6)

Out of 721 school going children, majority of participants, 541 (75%) belonged to the age group 16-19 years, more than half were girls 389 (54%) and belonged to urban locality 359 (51%)(Table 2).

**Table 2:** Socio-demographic characteristics and pattern of internet use among secondary school-going adolescents.

Characteristics	n (%)
Age group (in years)	
13-15	101 (14)
16-19	541 (75)
Above 19	79 (11)
Gender	
Boy	325 (45)
Girl	389 (54)
Not recorded	7 (1)
Place of residence	
Urban	359 (50)
Rural	362 (50)
Most preferred method internet access	
Smart phone	586 (81)
Tablet	9 (1)
Laptop	41 (06)
School computer	69 (10)
Internet café	13 (2)
Not recorded	3 (<1)
Use of smart phone for internet access	
Yes	388 (54)
No	333 (46)
Use of tablet for internet access	
Yes	72 (10)
No	649 (90)
Use of laptop for internet access	
Yes	240 (33)
No	481 (67)
Use of School Computer for internet	
access	
Yes	550 (76)
No	171 (24)
Use of internet cafe for internet assess	
Yes	179 (25)
No	542 (75)
Use of internet for education	
Yes	568 (79)
No	153 (21)
Use of internet for Social Networking	
Yes	402 (56)
No	319 (44)
Use of internet for gaming	
Yes	193 (27)
No	528 (73)
Internet Facility at home
Yes	622 (86)
No	99 (14)
Are parents aware of your internet use	
Yes	647 (90)
No	73 (10)
Missing	1 (<1)
Preferred time of use of internet	
During school hours	20 (3)
After school hours	341 (47)
At night	360 (50)
Preferred Place of internet use	
Café	38 (5)
Hostels	12 (2)
School computer lab	128 (18)
Residence	380 (53)
Others	158 (22)
Missing data	5 (1)
Use Internet in group or individually	
Group	73 (11)
Individual	645 (89)
Missing	3 (<1)

Around 622 (86%) respondents had internet facility at home. Smartphone was reported as the most preferred method of internet access by 586 (81%) students. More than half 388 (54%) actually use smart phone, another one-third 240 (33%) use laptop for internet access. A total of 568 (79%) respondents use internet for education; 402 (56%) for social networking and 193 (27%) for gaming. Most of them prefer to use internet after school hours 341 (47%) and at night 360 (50%). More than half 380 (53%) like to use internet at their homes, 645 (89%) use internet individually rather than in groups 73 (11%)(Table 1,2).

Internet addiction was found different in different age groups, gender and other sub-groups (Table 3).

**Table 3:** Internet addiction in different groups among secondary school-going adolescents.

Characteristics	Total	Internet Addicted n (%)
Age group (in years)		
13-15	80	30 (37)
16-19	465	199 (43)
Above 19	74	30 (40)
Gender		
Boy	289	123 (43)
Girl	323	133 (41)
Place of residence		
Urban	322	152 (47)
Rural	297	107 (36)
Internet Facility at home		
No	78	24 (31)
Yes	539	233 (43)
Are parents aware of your internet use No	63	21 (33)
Yes	556	238 (43)
Use smart phone for internet access		
		
No	280	108 (39)
Yes	339	152 (45)
Use of internet for education		
No	144	79 (55)
Yes	475	180 (38)
Use internet for social networking No	259	85 (33)
Yes	360	175 (48)
Preferred time of use		
At night	317	156 (49)
After School Hours	282	97 (34)
During school hours	18	6 (33)
Preferred place of use		
Café	35	14 (40)
Hostel	11	6 (55)
School Computer lab	101	32 (32)
Residence	326	147 (45)
Others	142	60 (42)

Qualitative results

Five overarching themes emerged from the in-depth interviews relating to: a) factors triggering internet use, b) internet-related activities, c) hazards and benefits of internet use, d) trying to reduce internet use, and e) parents complain about internet use.

Theme 1. Factors triggering internet use.

Subthemes included: a) boredom, b) when feel sleepy, c) internet activities, d) stress or anxiety, and e) peer pressure.

Many participants reported that boredom triggered their desire to use the internet. Students reported internet as one of their strategies for coping with boredom.

“I like to be online as I remain engaged. “–16 year old girl

Some even use internet when they feel sleepy in order to remain awake. For some, the strongest urges came with negative emotions such as stress or anxiety, both personal and school related. Desire to engage in specific internet activities such as chatting with friends, updates from friends, online shopping, gaming also led to internet use. Peer pressure was also reported to be one of the reasons triggering internet use.

“Friends pressurize to come online and chat.” –19 year boy

Theme 2. Internet related activities.

This theme describes the online activities participants’ favored and the reasons for their enjoyment of those activities. Many participants engaged in multiple activities while on the Internet.

Subthemes included: a) social media, b) school work, c) online shopping, d) gaming, and e) chatting with friends.

Most participants reported using some form of social media mainly Facebook. Due to the accessibility of social media sites on mobile devices, many participants noted their use as part of their daily routine.

*“I use Facebook messenger, for chatting and updating information. Look at new pictures and information. It is exciting to be online, gaming and chatting with friends, making new friends.”* -19 year old boy

The extent of daily use ranged from casual (e.g., *“I like sharing thoughts or ideas or moods with followers on Facebook, looking at updates from friends”*) *to compulsive (e.g., “It has become a habit, sometimes I spend the whole night doing it.”*)

Theme 3. Hazards and benefits of internet use.

Hazards of internet use have been categorized into the following sub-themes: a) health hazards, b) effects on social and personal life, and c) effects on academic performance.

Participants discussed adverse health consequences as an effect of internet overuse which included eye problems, fluctuations in mood, lack of sleep and compulsive behavior to use internet. Participants have also mentioned other behavioral problems such as,

“I tend to forget things.” -17 year old boy

“I am not able to have meals on time.” 17 year old boy

“In fact, I eat a lot when I am online.” 19 year old girl

Delays in submitting school assignments and poor academic performance have been reported to be due to excessive internet use.

*“I feel like I have wasted a lot of time, I am not able to get time for preparation during examination.”* -18 year boy

*“My parents say that my poor academic performance is due to excessive use of internet. I agree to what they say but I cannot stop using internet.”* -17 year old boy

*“Others often complain that I miss deadlines, I always procrastinate.”* -18 year old girl

A 19 year old boy pointed out that the negative effects of Internet overuse on his inability to focus.

*“I tend to forget other activities.”* -19 year old boy

He was also wary of other hazards of internet like exposure to harmful sites, fake accounts and online defamation.

*“I had a fight online once which put me off.”* -19 year old boy

When asked about the influence of internet use on social life, many participants responded by saying that it has reduced personal interaction with family members and friends.

*“Before using internet, I used to spend time with my parents, however, now I spend very little time with them. I am not even able to face other people and open up outside.”* -19 year old girl

*“I never go out, instead prefer to stay online.”* -19 year old girl

Reported benefits of internet use included: a) academic learning, b) solving school assignments, c) make new friends and keep in touch with old friends, c) get new ideas, and d) saves boredom.

Participants noted positive social effects of ilnternet use. Internet can facilitate easy networking with family members, friends, and community. It is also a useful resource for solving school assignments.

*“Internet helps in making new friends and building new relationships, it keeps us engaged.”* -16 year old girl

Theme 4. Barriers to reduce internet use.

Furthermore, participants had difficulties in trying to reduce internet use, with some attributing it to the addictive nature of it and the compulsion to be online.

One of the participant even said that *“I cannot stay without using it.”* –18 year old boy

*“Tried reducing internet use but failed as going offline makes me feel like missing something in life.”* –18 year old girl

*“I tried during the examination, but I failed because I am so used to it.” *–17 year old boy

*“A part of me is missing when I am not online.”* –19 year old girl

For many participants, time spent on the ilnternet was more difficult to control and ended up spending the whole night using internet.

*“I spend whole night in one continuous session of use.”* –16 year old girl

*“I plan to remain online for few minutes, but end up staying much longer than expected.”* –18 year old girl

Theme 5. Parents complain about internet use.

Participants said that parents usually complain about their internet use because they are concerned about their studies. For some it was quite irritating, whereas others reported fighting with the parents over this matter.

One participant even mentioned that *“no complaint from my parents so far as I use secretly.*“ –19 year old girl.

*“Parents tell to study rather than using internet. I feel irritating sometimes.”* –19 year old girl

*“Yes, I fight with my mother most of the time regarding internet use.”* –17 year old girl

*“I agree to what they say but however I cannot stop using internet.”* –18 year old boy

## DISCUSSION

This is the first study in Bhutan highlighting the problem of internet addiction and other psychological co-morbidities among school-going adolescents. The key findings of the study are: a) one out of every three four out of 10 adolescents are addicted to internet, b) depression and anxiety were the associated psychological co-morbidities among internet addicts, c) high IA was found among those with internet facility at home, use of smartphone for internet access, use of internet for social networking and night time use of internet were associated with IA, d) addictive nature of internet and compulsion to be online were the barriers to reduce internet use, e) boredom, stress/anxiety and peer pressure were found to be the key triggers of internet use, and f) IA was found to affect academic performance and lead to sleeplessness.

The present study reported the prevalence of IA among secondary school-going adolescents to be 34.40%. Other recent studies done in the Asian region using the same tool have reported varying prevalence estimates ranging from 6.7% to 36.9%. The wide variation in the results of similar studies done in this region since 2010 could be attributed to the different time periods when they were conducted, slightly varying age groups, and different internet penetration rates, unique cultural and social contexts.

The present study showed that depression and anxiety scores were significantly higher among internet addicts. This association is in line with the findings reported in previous studies.^7,12-19^ One of the possible reasons for the associated psychological co-morbidities is the disrupted sleep-wake cycle and a higher rate of insomnia among heavy internet users.^19^ In this study, the participants reported night time as the preferred time of internet use and some of them even used to spend the whole night online which substantiates the plausible association above. This study also found that internet users avoid interpersonal relationships with real and known people as they prefer staying online (*“I am not even able to face other people and open up outside.*“ –19 year old girl) which might be another reason for the associated psychological morbidities as supported by other studies.^20^

In this study, respondents reported “boredom” as another trigger for internet use. Other studies also echoed the finding which reported internet use to kill time.^21,22^ When adolescents are bored or dissatisfied with their leisure time, they may be motivated to seek excitement and pleasure from cyberspace. This has important policy implications. Participation in leisure time outdoor activities has been shown to improve academic performance and lower stress levels.^23^ Thus, it is important to encourage students to engage in outdoor activities may it be sports, social gatherings or family functions rather than spending too much time on the Internet. Parents and school teachers, both have a crucial role to play in creating an enabling environment for the same.

The adolescents reported that parents used to complain about their internet use which they did not like. (*“Parents tell to study rather than using internet. I feel irritating sometimes*.” –19 year old girl). Although parental monitoring has been found to be effective in reducing internet use, we suggest a more educative approach rather than a regulatory approach.^24^

Many adolescents reported that it is difficult to reduce internet use due to its addictive nature. A review paper by Greydanus, et al. also highlighted the addictive nature of internet use among adolescents.^16^ It is postulated that adolescents seek high-risk and novelty-seeking experiences of emotional intensity and liability which makes them vulnerable to various addictions.^25^ IA was higher among adolescents using smartphone.^26^ Smartphone is a handy, convenient device to connect to the internet and it is possible that a smartphone user will use internet in situations of boredom. The adolescents stated that they had problem in submitting the schools assignments and preparing for exams leading to overall poor academic performance. This conforms well with the findings reported by other studies in adolescents.^12,27,28^ Earlier studies also show that excessive internet use leads to sleep deprivation and irregular sleep-wake pattern which has shown to be linked to poor academic achievement and other psychological co-morbidities.^19,29^

Comprehensive education should be given to adolescents from their early years about not only the benefits but also the potential adverseissues about the internet. Adolescents should be educated about the need for controlled and educated use of this technology. Engagement in outdoor activities should be promoted in schools and homes. The role of parents is also crucial in monitoring child’s internet use and educating them about the hazards of internet use; enforcement may not work here.

The study demonstrates that IA is a common phenomenon among students with a potential negative impact that needs to be managed in the schools. The school counselors should be adequately trained to identify warning signs, screen, and counsel and refer to trained specialists if required through a structured referral mechanism.

The study used a nationally representative sample of school-going adolescents which makes the findings generalizable to the whole country. The survey achieved a 100% response rate due to the support of the Ministry of Education and the school staff. Validated tools were used to measure domains such as internet addiction, depression, anxiety and stress. The study also adhered to the Strengthening the Reporting of Observational Studies in Epidemiology (STROBE) and COREQ guidelines in reporting the quantitative and qualitative findings of the study respectively.^11,30^

There were some limitations in the study as well. This being a cross-sectional study, causal relationship between IA and co morbid psychiatric disorders cannot be established. Data collected in this study were self-reported which might induce social desirability bias. The IDIs were conducted with the adolescents in the school which might have influenced their answers. However, it was ensured that the teacher was not present in any of the interviews to reduce the response bias. The study did not interview the parents, caregivers and teachers, which could have given a different perspective on the use of internet. Future qualitative research could be planned to get the views of these key stakeholders.

As internet addiction lacked definite clinical diagnostic criteria, further research could also be conducted to precisely measure IA taking into account various related factors identified in this study. Further, intervention studies should be designed to establish effective preventive and management strategies to reduce IA among this vulnerable group.

## CONCLUSIONS

Prevalence of internet addiction and other psychological co-morbidities among school-going adolescents is high compared to standard data and concerning. The factors identified to be associated with IA and the user’s perspective on internet use provides valuable information in designing preventive interventions. The role of teachers and parents is crucial in monitoring child’s internet use and educating them about the hazards of internet use through a structured curriculum.
